# Hepatitis C Care Cascade in Opioid-Dependent Emergency Department Patients

**DOI:** 10.5811/westjem.47232

**Published:** 2026-08-06

**Authors:** Jordi Garcia-Diaz, Whitney Covington Taylor, Lauren A Walter

**Affiliations:** University of Alabama at Birmingham, Heersink School of Medicine, Department of Emergency Medicine, Birmingham, Alabama

## Abstract

**Introduction:**

Emergency departments (ED) have become important venues for hepatitis C (HCV) screening. Similarly, EDs have had increasing engagement in the management and treatment of opioid use disorder (OUD). Persons with OUD are at disproportionate risk for acquiring HCV. We sought to assess the intersection between OUD and HCV care through observing the uptake of HCV screening and subsequent steps in the HCV care cascade in an ED-OUD cohort.

**Methods:**

This retrospective cohort study analyzed patients enrolled in an ED-OUD treatment program as a convenience sample subgroup of a larger institutional ED opt-out HCV screening program conducted at a single urban site in Alabama between July 2019–June 2022. Opt-out, nonfocused HCV screening provision was standardized at triage, excluding pregnant and incarcerated individuals. Descriptive analyses were conducted to ascertain screening uptake, positive screening, confirmatory testing, linkage to HCV treatment centers, and initiation of antiviral therapy. Additional patient level demographics considered included sex, race, age, insurance status, and domicile status.

**Results:**

Of the approximately 75,000 patients screened annually across the study timeframe, July 2019–June 2022, 359 patients with OUD were enrolled; enrolled participants were more likely to be male (64.9%), white (77.4%), aged 25 to 44 (73.3%), uninsured (80.5%) and housed (80.5%). Only 99 (27.6%) received an HCV antibody (Ab) screening test, 52 (52.5%) of which were positive. Thirty-three patients (63.5%) of the Ab-positive cohort were confirmed HCV viremic or 9.2% of the entire OUD cohort. Three (9.1%) of those with active viremia had documented post-ED HCV-specific care and only one was initiated on direct-acting antiviral therapy. White patients were more likely to be Ab-positive as compared to Black patients (88.5% vs 9.6%); however, Black patients were more likely to have active viremia (15.2%). Antibody-positive females had higher rates of viremia (80.0%) compared to Ab-positive males (53.1%).

**Conclusion:**

Screening capture, subsequent RNA-positive linkage to care, and medication initiation rates were suboptimal, revealing opportunities for improvement across the care continuum. Incidence of active HCV viremia in the ED-OUD patient cohort is nine times higher than that in the general population and three times higher than that of the local general ED cohort.

## INTRODUCTION

Persons with opioid use disorder (OUD) face a significantly elevated risk of contracting hepatitis C virus (HCV) due to various behavioral and systemic factors.^1^ Injection drug use (IDU), particularly the sharing of needles and other paraphernalia among individuals with OUD, constitutes the primary mode of HCV transmission via direct blood-to-blood contact.^1^ According to the Centers for Disease Control and Prevention (CDC), IDU accounts for approximately two-thirds of new HCV infections in the United States (U.S.).^2^ Furthermore, individuals with OUD are more likely to engage in high-risk behaviors such as unprotected sex, which can further contribute to the transmission of HCV.^3^ Moreover, the structural vulnerabilities faced by individuals with OUD, such as limited access to healthcare, socioeconomic disparities, and stigma, can impede their ability to access HCV prevention, testing, and treatment services, often referred to as the hepatitis C Cascade of Care–similar to that of human immunodeficiency virus (HIV) treatment programs.^4^,^5^ Disruptions in this series of interventions leads to a reduction in success of HCV treatment to sustained virologic response.^4^

Although the United States Preventive Services Task Force (USPSTF) recommended HCV screening and treatment in primary care settings, emergency departments (ED) have emerged as critical venues for HCV screening.^6^–^8^ Emergency departments have the potential to reach a diverse and often underserved patient population, making them an essential component of efforts to expand access to testing and linkage to care.^6^,^7^ This shift aligns with the World Health Organization’s (WHO) goal of eliminating HCV as a public health threat by 2030, emphasizing the need for widespread screening and treatment initiatives beyond traditional healthcare settings.^9^ Research indicates that a substantial proportion of individuals with HCV infection remain undiagnosed, and many of them frequently utilize emergency healthcare services.^10^,^11^ Studies have shown that implementing routine HCV screening programs in EDs can effectively identify undiagnosed cases and link patients to appropriate care and treatment services.^10^,^12^,^13^ As EDs expand their role in managing OUD by identifying individuals with OUD, initiating medication for OUD (MOUD), and linking patients to treatment, emergency physicians are uniquely positioned to address coexisting health concerns like HCV by engaging patients across the HCV care cascade.^14^–^16^

The HCV care cascade represents a series of sequential steps essential for effective HCV management: initial screening to identify infected individuals; confirmatory testing with HCV RNA to distinguish active from resolved infections; linkage to specialized care for treatment planning, initiation of direct-acting antiviral therapy; and sustained virologic response achievement indicating cure.^17^ Each step presents potential barriers where patients may be lost to follow-up, from initial screening uptake through treatment completion. Emergency departments offer a unique opportunity to engage high-risk populations who may not access routine healthcare, potentially capturing patients early in this cascade and facilitating progression through subsequent care stages.^18^ Linkage to a first appointment, however, does not ensure progression through subsequent cascade steps, and understanding attrition rates at each downstream stage is therefore critical for emergency physicians and public health officials seeking to design referral partnerships and supportive services capable of addressing the full continuum of care. For individuals with OUD who face elevated HCV risk due to IDU and often experience fragmented healthcare access, the ED encounter may represent a critical intervention point to initiate or re-engage patients in the HCV care continuum.

**Population Health Research Capsule**

What do we already know about this issue?

*Persons with opioid-use disorder (OUD) carry disproportionate hepatitis C virus (HCV) risk. Emergency departments (ED) are emerging screening venues, yet persons with OUD remain underscreened in these settings*.

What was the research question?

*What is HCV screening uptake and care cascade completion among ED patients enrolled in a medication for OUD program?*

What was the major finding of the study?

*Only 27.6% of OUD patients were HCV-screened; 9.2% had active viremia, approximately nine-fold greater than the general population*.

How does this improve population health?

*Revealing care cascade gaps in high-risk OUD populations informs targeted ED-based interventions to reduce HCV burden, notably in non-Medicaid expansion states*.

Prior studies have identified the ED as an important “crossroads of intersecting epidemics,” namely OUD and HCV; however, a recent analysis by Lyons et al focused on this overlap that included ED visits from 2017–2018, noted that ED patients with OUD are underscreened for HCV in this setting.^19^,^20^ The authors of this report concluded that HCV screening in EDs was uncommon among patients with OUD, including individuals who engage in IDU, even in EDs with dedicated screening initiatives. Since this prior analysis, the COVID-19 pandemic has exacerbated the opioid epidemic and the incidence of new HCV infections in the US has continued to rise.^21^ Importantly, prior analyses focused primarily on screening uptake rather than the complete HCV care cascade, and none have characterized cascade completion in a non-Medicaid expansion state where structural barriers to treatment are more pronounced. To date, no studies have assessed the rates of completion for each step of the HCV care cascade among patients who are initiated on MOUD in the ED, particularly in the Southeastern U.S., an often under-resourced geographic area of the U.S. Our goal was to assess the uptake of HCV screening and subsequent HCV Care Cascade among a convenience sample of ED patients with OUD who were enrolled in an ED-based MOUD program. The results of this study can help inform local public health officials into which targeted or wrap-around services may be necessary to end the HCV epidemic by 2030 as proposed by the WHO.^9^

## METHODS

### Study Design and Protocol

This retrospective cohort study is a convenience-sample subgroup analysis drawn from a previously described ED-based MOUD initiation and treatment referral program at a single site in Alabama. The program, which has been extensively described in the literature, enrolled patients presenting to the ED with opioid overdose, withdrawal, or detoxification requests who met Diagnostic and Statistical Manual of Mental Disorders (DSM-5) criteria for moderate-to-severe OUD and received treatment in the ED.^22^,^23^ The current analysis examines HCV screening uptake and care cascade outcomes among patients enrolled in the ED-MOUD program (n = 359) during the study timeframe (July 2019–June 2022), using the ED-based universal opt-out HCV screening program as a parallel data source.^22^,^23^

### Emergency Department-Medication for Opioid Use Disorder Program

Patients presenting to the ED with a primary complaint of nonfatal opioid overdose, opioid withdrawal, requesting opioid detoxification, or otherwise meeting DSM-5 criteria for moderate-to-severe OUD, were considered eligible for program inclusion, following medical stabilization and clearance. Patients enrolled in the ED-MOUD program, following medical stabilization and clearance, were eligible to begin MOUD (buprenorphine/naloxone) initiation in the ED (for those with Clinical Opiate Withdrawal Scale score > 12) and/or a prescription. They were also subsequently referred for definitive OUD treatment.^23^

### Hepatitis C Virus Screening Program

Parallel to the OUD-focused program, the ED has an HCV screening and linkage program with funding provided by Frontlines of Communities in the United States (FOCUS) Program, Gilead Sciences, Inc. [PI: LAW], which supports HIV, HCV, and HBV screening and linkage to a first appointment. FOCUS partners do not use FOCUS awards for activities beyond linkage to a first appointment. The Department of Emergency Medicine [PI: LAW] is supported by a grant [1H79TI081609] from the Substance Abuse and Mental Health Services Administration (SAMHSA). This protocol has also been previously extensively described^24^; in summary, all ED patients aged 18–75 years without an HCV test result available in the electronic medical record, Cerner Health Records (Cerner, North Kansas City, MO), within the prior 12 months were eligible for HCV testing. The institution’s electronic health record (EHR) is not connected to outside hospital systems, and therefore HCV test results from external institutions were not accessible and could not be used to assess eligibility for the screening program. Patients were notified of the nontargeted HCV Ab testing program by nursing staff in triage in an opt-out fashion. It is standard protocol that nursing staff ask patients during triage if they carry a diagnosis of HCV or HIV. The onus is on the patient to request not to be tested, and if not, an order will be placed for HCV and HIV screening. Additionally, those who have a positive HCV testing result in the past year were excluded from the analysis. Those with known diagnosis were then asked if they have been treated, and if so, they were not screened. Patients could decline for any reason (current infection or previously treated, uninformed on their high-risk nature, refusing blood draw, perceived associated stigma with HCV, etc). Testing was performed via a venous blood draw. Hepatitis C virus Ab screening test results were typically available within approximately one hour after venipuncture, and therefore most patients remained in the ED at the time of Ab result. If a blood specimen was not obtained, then this was considered as a missed screening opportunity. Historically, HCV screening at the institution is 20% on average.^13^,^25^

Patients with positive HCV Ab testing results discussed with their provider regarding the positive results, the possibility of treatment, and the linkage to care plan. Providers confirmed patients’ phone numbers (as well as a secondary contact to reach patients) and provided patients packets with written instructions with more information regarding HCV screening, next steps, and further care. Following a positive HCV Ab screening, a confirmatory reflex HCV RNA confirmatory test was sent using the previously obtained blood sample with results available within 1–2 days, on average.^25^ Patients were then subsequently contacted by a linkage-to-care coordinator, who calls the patient once the RNA result is available and facilitates the linkage to first appointment with an HCV treating provider, consistent with the scope of the FOCUS program funding. If after seven callback attempts at different times and days, and if there was no evidence of patient linking to care on electronic medical record review, the patient was considered to have failed to link to care. Patients attending their first appointment were considered to be a successful linkage-to-care. Pregnant and incarcerated patients were excluded from this analysis.

The institutional review board approved all study procedures, and funding agencies were not involved in study design, analysis, drafting of the manuscript, or the decision to submit for publication. STROBE (Strengthening the Reporting of Observational studies in Epidemiology) guidelines were used to conduct and compose this review.^26^ In line with methodologic standards for medical review, a single author (LW) independently reviewed deidentified, aggregated patients from Cerner Health Records that met inclusion and exclusion criteria that were pre-established and described above.^27^ The single abstractor (LW), was a part of the design of the project, therefore did not require training. Additionally, the reviewer did not require abstraction forms nor monitoring, and they were aware of the hypothesis and study objective. Since only one reviewer was involved, inter-rater reliability was not applicable.^27^

### Study Setting and Population

The University of Alabama at Birmingham Medical Center is an academic tertiary care medical center located in an urban area serving a population of more than 1.1 million people. Alabama is a non-Medicaid expansion state, meaning it has not adopted the Affordable Care Act’s provision allowing states to extend the Medicaid eligibility to adults earning up to 138% of the federal poverty line. This leaves a significant portion of the population uninsured (17.5%), nearly twice the rate of Medicaid expansion states, with limited options for healthcare access.^28^

### Data Collection

Research staff enrolled participants in the ED MOUD program, contingent on staff availability (typically from 09:00–00:00, seven days per week). During enrollment, they obtained and recorded demographic data, including race, sex, age, domicile status and insurance status. Domicile status was classified as “currently housed,” to include private home, apartment, or trailer, versus “experiencing homelessness” (sheltered or unsheltered). Insurance status was categorized as private insurance, public insurance (Medicaid and/or Medicare), or “self-pay.” Same-visit ED HIV and HCV screening uptake and results were also recorded. Electronic health record chart review was conducted to assess documented engagement in HIV or HCV treatment following ED MOUD program enrollment, with the objective of evaluating completion of post-ED follow up. Provider documentation and/or laboratory results (eg, HCV RNA quantitative levels, etc) were considered. At time of ED MOUD program enrollment, research staff also recorded participant self-reported IDU history, categorized as current/active, within the last six months, greater than six months, or never. All study data were collected and managed by research staff using Research Electronic Data Capture (REDCap), a secure, web-based data capture application.^29^ The data was defined by a multiclass classification, with well-defined, categorical outcomes (eg, referral, screening, and treatment initiation, age categories, identified race, sex assigned at birth) that minimized potential bias and minimized subjectivity.

### Statistical Analysis

Descriptive statistics were utilized to compare testing uptake and outcomes by patient demographics. Data responses that consisted of “don’t know,” “declined to answer,” or missing were excluded from statistical analysis. Data analysis was conducted using International Business Machines (IBM) Statistical Package for the Social Science (SPSS) for Windows, version 27 (IBM Corp., Armonk, N.Y., USA). There was no formal power analysis, as sample size was limited to patients enrolled in the ED MOUD program during the study timeframe. Given the descriptive aims of this secondary analysis, no sensitivity analyses were performed.

## RESULTS

During the study period, approximately 75,000 patients checked into the emergency department annually. Of these, 359 patients were enrolled in the ED MOUD program, and 27.6% (n = 99) received an HCV screening test compared with the average screening rate of 20% among patients without MOUD. Participants were more likely to be male (64.9%), white (77.4%), aged 25 to 44 (73.3%), uninsured (80.5%) and housed (80.5%) (Figure, Table).

While the majority (82.5%) declined to answer, 15.0% (n = 54) of participants reported IDU within six months prior to enrollment, and 9.2% (n = 33) reported active IDU. Screening uptake was low, and substantial attrition occurred at each subsequent step of the HCV care cascade. Among participants with self-reported IDU within six months of enrollment, 71.4% were not tested for HCV. Of the tested IDU subgroup, 64.3% (n = 9) were Ab positive and 28.6% (n = 4) were confirmed HCV viremic. 20.4% (n = 75) of patients were previously aware of HCV diagnosis, and none have preciously sought care. Only 18.2% (n = 6) viremic patients self-reported a previous HCV diagnosis on our questionnaire.

White patients were more likely to be Ab-positive as compared to Black patients (88.5% vs 9.6%); however, Black patients were more likely to have active viremia, representing 15.2% of RNA-positive patients despite comprising only 9.6% of Ab-positive patients. Among Ab-positive patients, females represented 38.5% of those tested, but accounted for a disproportionate amount for RNA positive cases (48.5%), suggesting higher rates of active viremia compared to Ab-positive males (80.0% vs 53.1%). Post-ED HCV-specific care was only found for three patients (9.1%) with active viremia; only one was initiated on direct-acting antiviral therapy (with no documentation of subsequent sustained virologic response).

## DISCUSSION

Screening rates in this OUD cohort were comparable (27.6%) to nontargeted testing uptake in the general ED population at this site during the study timeframe (20% of the total average ED volume).^26^ Although this similarity suggests that the opt-out protocol reached patients in the MOUD protocol at similar rates to the general population, it underscores a critical gap that patients with OUD are at a disproportionately higher risk of HCV yet were screened at no greater a rate than the general population. Active HCV viremia was identified in 9.2% of the whole study cohort, approximately nine times the rate in the general population (1.0%) and three times the positivity rate of the general ED population at this site. Recurrent exposure related to substance misuse and lack of prior available treatment options likely explain this significant difference. Several barriers specific to this population may account for this failure to achieve meaningfully higher screening uptake and subsequent steps of the HCV care cascade, including HCV-related stigma, low perceived utility of testing, and structural treatment barriers.

Hepatitis C virus remains highly stigmatized, and patients who present to the ED may seek to avoid labeling or other potential perceptions when consenting to HCV testing.^30^ Prior studies have demonstrated that individual awareness of HCV risk and exposure is often lacking and nearly one-third of individuals with HCV are unaware of their infection status.^31^,^32^ Active substance misuse has previously been demonstrated to be an independent barrier to HCV treatment linkage.^24^ In 2019, Jost et al published the results of qualitative interviews of persons who inject drugs (PWID), highlighting a recurrent tendency in this cohort to downplay the risk and possibility of acquiring HCV.^33^ Furthermore, for PWID in particular, knowing one’s HCV status may have been perceived as providing limited value.^34^ Notably, during the study timeframe, Alabama Medicaid sobriety requirements mandated six months of sobriety prior to HCV treatment initiation, a policy not lifted until October 2022, potentially impacting nearly a quarter of this study’s viremic cohort.^35^ Furthermore, for the infected individuals who are self-pay, nearly 70% in this study, the cost of direct-acting antiviral therapy may make obtaining treatment extremely prohibitive, and limits patients’ ability to access treatment.^36^,^37^ Individual ongoing substance misuse and/or one’s financial situation may make patients question the personal utility of HCV testing, despite prior demonstrations of cost-effectiveness from a health system and population health perspective.^38^

This study addresses key gaps in the existing literature. Prior analyses of HCV screening in ED-based OUD populations have focused primarily on screening uptake rather than full cascade completion.^19^,^20^ Additionally, to our knowledge, none have characterized cascade outcomes in a non-Medicaid expansion state, where structural barriers to treatment are more pronounced.^28^,^35^ By capturing the complete HCV cascade within this high-risk ED-based OUD cohort, this study provides a more comprehensive picture of cascade attrition in a structurally under-resourced setting.

Among patients diagnosed with HCV in the ED, 81.8% were found to be viremic, demonstrating high capture of new infections. These findings highlight the importance of the ED as a screening venue, particularly for this population, rather than in the outpatient addiction setting. While outpatient addiction medicine clinics represent a natural venue for HCV screening given their OUD-focused patient population, attendance at these clinics following ED referral is inconsistent, with studies of ED-initiated MOUD programs demonstrating that a significant proportion of patients do not follow up, making the ED encounter the most reliable and often only guaranteed point of contact for this population.^39^,^40^ When programs have involved social work and patient care navigators for ED-based screening and treatment for similar infections, such as HIV, viral suppression rates of greater than 50% are achieved, suggesting this model may be transferable to HCV in OUD populations.^41^ The ED frequently acts as the initial and main connection point to the healthcare system for individuals with OUD, as they are unable to overcome routine healthcare access limitations.^42^,^43^ To underscore this, nearly one in five in this study were experiencing homelessness at the time of their ED visit.^44^ Current ED support staff such as peer navigators, who often assist with the referral to OUD treatment centers, could naturally expand their scope to include HCV education and testing encouragement prior to discharge.^45^ Studies looking at post-overdose care services in the ED suggest that structured follow-up interventions improve subsequent treatment engagement, supporting the need for similarly structured HCV screening protocols within MOUD programs.^39^,^40^ Deferring HCV screening to a clinic appointment risks losing a critical intervention opportunity in the patients who may never arrive.

Addressing these barriers in the HCV care cascade requires both system-level and patient-level interventions supported by emerging evidence in ED-OUD populations. Patients in this cohort may present to the ED with complaints that do not typically require venous blood sample as a part of their workup, making HCV screening orders less likely to be prioritized. Incorporation of an HCV fingerstick test in the future may increase testing uptake, as patients who might decline screening via venipuncture may be more willing to undergo a fingerstick blood draw based on patient preferences.^46^ Additionally, despite a turnaround time of one hour, a point-of-care test will likely further increase medical professionals recommending this screening test is performed before discharge. This less-invasive, single-step method has been shown to be practical in primary care settings outside the U.S.^47^ If made available in the U.S., this method may improve testing uptake in this cohort in the general ED setting. Concurrently, providing healthcare staff with education and training on the importance of HCV testing, stressing its high prevalence among individuals with OUD, and strategies for offering and encouraging testing in a nonjudgmental manner represents a key complementary intervention.^48^,^49^

Future studies should evaluate whether the implementation of point-of-care HCV testing within ED workflows, which would provide rapid result availability and reduced procedural burden, can meaningfully improve screening uptake and HCV care cascade completion in this population. Additional studies considering broader, patient-targeted campaigns to reduce the perceived stigma associated with both OUD and HCV infection may help promote a supportive and non-discriminatory healthcare environment resulting in fewer patient opt-outs.^50^

Beyond screening and care cascade gaps, the present study also identified disparities in HCV outcomes across demographic subgroups that warrant attention. Although interpretation is fairly limited by small subgroups, the racial disparity correlates with prior observational studies which note Black patients are less likely than White patients to be prescribed treatment for HCV.^51^ This might also reflect the recent demographic shift in the opioid epidemic which is increasingly impacting Black patients.^52^ It may be that this subgroup has not had the same chronologic exposure to HCV testing or treatment referral follow-up.^52^ Women have also been shown to be less likely to receive HCV treatment which this study reiterates.^53^,^54^ These disparities underscore the important role the ED could play, as a critical intervention point for Black patients and women with OUD, given their reduced likelihood of accessing HCV care through traditional healthcare pathways. Equity-informed approaches to ED-based screening and linkage are warranted.

## LIMITATIONS

This analysis was limited to a single site and therefore, results may not be generalizable to all sites. The cohort included OUD participants enrolled in an ED-initiated treatment program and therefore excluded many patients with OUD who presented for other reasons other than overdose, withdrawal, or detoxification, introducing the potential for selection bias. However, this patient population likely represents a moderate-to-severe OUD subset of people who may inject drugs. Furthermore, since this was a retrospective observational study, the presence of other substance use disorders or high-risk behaviors beyond those captured in the medical record cannot be excluded as potential confounders. Patients with a positive HCV result in the institution’s lab within the past 12 months were automatically excluded from the screening. Additionally, there is a limitation in the ability of the EHR to see HCV testing results performed at outside institutions. The modest sample size limits broader interpretation, however, it is itself reflective of the study’s central finding: poor completion rates across the HCV care cascade. Future studies with larger cohorts are needed to confirm these findings. Missing data was limited to race and insurance status, which can limit generalizability, but this was secondary demographic data and does not take away from the underlying message behind the care cascade.

## CONCLUSION

Active HCV viremia was identified at rates approximately nine times that of the general population in this ED-based OUD cohort; yet, screening uptake was poor, and completion of the HCV care cascade was correspondingly low, with only three RNA-positive patients linked to care, and only one initiated direct-acting antiviral therapy. These findings highlight the gap in the HCV care cascade in this high-risk population and underscore the need for targeted interventions to improve steps of the HCV care cascade particularly in non-Medicaid expanded states. Future studies evaluating point-of-care HCV testing and peer navigator-facilitated advocacy within ED-based MOUD programs could determine if these targeted interventions can improve cascade completion in this high-risk population.

## Figures and Tables

**Figure:**
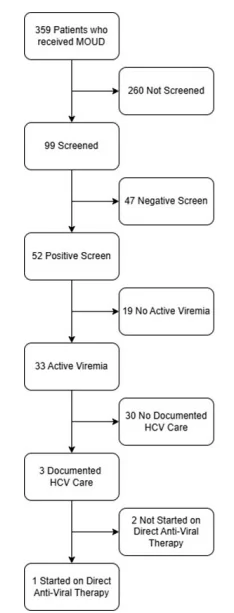
Sampling Flow Diagram. Summary of patients who received medications for OUD, if they were screened, how many found to be actively viremic, how many were linked to care, and how many were started on direct-acting anti-viral therapy. *HCV*, hepatitis C virus; *OUD*, opioid-use disorder.

**Table:** Hepatitis C virus testing uptake and outcomes among emergency department medication for opioid use disorder participants.

Demographic	All OUD Participants n = 359 n (%)	Tested n = 99 (27.6%) n (%)	HCV Ab+ n = 52 (52.5%) n (%)	HCV RNA+ n = 33 (63.5%) n (%)
Sex				
Male	233 (64.9)	57 (57.6)	32 (61.5)	17 (51.5)
Female	126 (35.1)	42 (42.4)	20 (38.5)	16 (48.5)
Race ^				
White	278 (77.4)	81 (81.8)	46 (88.5)	28 (84.8)
Black	65 (18.1)	15 (15.2)	5 (9.6)	5 (15.2)
Other	9 (2.5)	2 (2.0)	1 (1.9)	-
Age				
18–24	31 (8.6)	10 (10.1)	2 (3.8)	1 (3.0)
25–34	112 (31.2)	33 (33.3)	16 (30.8)	10 (30.3)
35–44	151 (42.1)	41 (41.4)	27 (51.9)	17 (51.5)
45–54	42 (11.7)	8 (8.1)	5 (9.6)	3 (9.1)
55–64	18 (5.0)	6 (6.1)	2 (3.8)	2 (6.1)
65–74	5 (1.4)	1 (1.0)	-	-
Insurance *				
Public	54 (15.0)	15 (15.2)	8 (15.4)	8 (24.2)
Private	31 (8.6)	9 (9.1)	1 (19.2)	1 (3.0)
Self-Pay	263 (73.3)	70 (70.7)	41 (78.8)	23 (69.7)
Domicile Status				
Undomiciled	69 (19.2)	21 (21.2)	14 (26.9)	10 (30.3)
Housed	289 (80.5)	78 (78.8)	38 (73.1)	23 (69.7)

Summary of patients who received medication for opioid use disorder in the emergency department from June 2019–June 2022, analyzed by sex, race, age, insurance status, domicile status.

* 11 missing data,

^ 7 missing data

*Ab+*, positive antibody; *ED*, emergency department; *HCV*, hepatitis C virus; *MOUD*, medications for opioid use disorder; *OUD*, opioid use disorder; *RNA*, ribonucleic acid.
